# AFM and FluidFM Technologies: Recent Applications in Molecular and Cellular Biology

**DOI:** 10.1155/2018/7801274

**Published:** 2018-07-04

**Authors:** Mohamed Yassine Amarouch, Jaouad El Hilaly, Driss Mazouzi

**Affiliations:** ^1^Materials, Natural Substances, Environment and Modeling Laboratory, Multidisciplinary Faculty of Taza, University Sidi Mohammed Ben Abdellah, Fez, Morocco; ^2^Biology, Environment & Health Team, Department of Biology, Faculty of Sciences and Techniques Errachidia, University of Moulay Ismaïl Meknes, Meknes, Morocco; ^3^Regional Institute of Education and Training Careers, Department of Life and Earth Sciences, Fez, Morocco

## Abstract

Atomic force microscopy (AFM) is a widely used imaging technique in material sciences. After becoming a standard surface-imaging tool, AFM has been proven to be useful in addressing several biological issues such as the characterization of cell organelles, quantification of DNA-protein interactions, cell adhesion forces, and electromechanical properties of living cells. AFM technique has undergone many successful improvements since its invention, including fluidic force microscopy (FluidFM), which combines conventional AFM with microchanneled cantilevers for local liquid dispensing. This technology permitted to overcome challenges linked to single-cell analyses. Indeed, FluidFM allows isolation and injection of single cells, force-controlled patch clamping of beating cardiac cells, serial weighting of micro-objects, and single-cell extraction for molecular analyses. This work aims to review the recent studies of AFM implementation in molecular and cellular biology.

## 1. Introduction

Atomic force microscopy (AFM) is a widely used tool in material science. It comes as an evolution of the scanning tunnelling microscope (STM) [1], which is restricted to electrically conductive materials. In this sense, AFM allows obtaining atomic-resolved images of insulators [2].

The sample is scanned by a pyramidal tip, which is set on a flexible cantilever spring fixed to an *x*-*y*-*z* piezo. The deflection of the cantilever is monitored with a laser beam reflecting the variation of the interaction forces between the tip and the sample surface. Thus, a topographic image of the sample is obtained by plotting the deflection of the cantilever versus its position on the sample, while the tip is scanning over the surface.

After becoming a standard surface-imaging tool, AFM was increasingly used in biological research to investigate many properties of living cells such as electromechanical [3] and cell adhesion properties [4]. Moreover, thanks to its compatibility with aqueous environments, AFM is considered as one of the foremost tool for monitoring microbes in real time [5]. In addition, AFM provides the possibility to study biological samples under physiological conditions without extensive cell preparation.

Since its development in 1986 by Binnig et al. [2], AFM technique has known many improvements; the fluidic force microscopy (FluidFM) represents one of them. Developed by Zambelli's group (ETH Zurich, Switzerland), FluidFM is an atomic force microscope which combines a conventional AFM with microchanneled cantilevers for local liquid dispensing via a fluidic circuit [6]. Using this technology, several single-cell biology challenges were addressed. Indeed, FluidFM allowed the isolation of single cells [7], quantification of bacterial adhesion forces [8], force-controlled patch clamping of beating cardiac cells [9], serial weighting of microobjects [10], and single-cell extraction for molecular analyses [11].

This review aims is to give an overview of the recent studies using the AFM technology in the molecular and cellular biology fields and presents an update of FluidFM technology usages in biological sciences.

## 2. AFM Principle

As mentioned above, atomic force microscopy is considered as an extension of the STM technique. The difference between these two approaches is based on the interaction type between probe and substrate. Indeed, when the probe interacts with the substrate via tunnelling current in the STM, AFM probes interact with the sample via forces. These forces can be roughly divided into attractive or repulsive forces.

**Figure 1:**
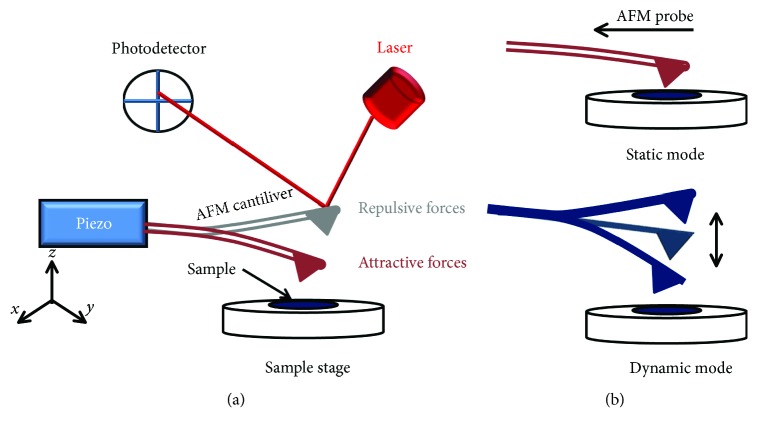
Atomic force microscopy (AFM). (a) Schematic representation of the AFM. (b) Operation modes of AFM.

A basic AFM setup includes a sharp pyramidal tip set on a flexible cantilever, which is mounted on electrical piezo (Figure 1). During the approach phase, the sample surface interacts with the tip via *Van Der Waals* forces. The attractive forces induce a deflection of the cantilever towards the sample surface. However, when the cantilever makes contact with the surface, an increasingly repulsive force repulses the cantilever from the surface. This reaction is known as *Pauli repulsion.* The cantilever deflection modifies the direction of the laser beam reflected from the backside of the cantilever, which *in fine* enables a very accurate measurement of the deflection. This detection method is called optical beam detection [12].

AFM operates under static or dynamic mode (Figure 1). In the former mode, termed DC-mode, the measured cantilever deflection is kept constant. Indeed, its height position is kept fixed or controlled by feedback controller. However, in the dynamic mode, also known as intermittent-contact mode, or alternating contact mode (AC mode), the probe is oscillated at a user-defined amplitude and frequency close to the resonance, while the tip-sample interaction affects the resonance frequency, the amplitude, and the phase of the oscillation. This mode offers the possibility to obtain more gentle topography information because of the bigger tip-surface separation (i.e., noncontact regime).

## 3. AFM Applications in Biology

### 3.1. DNA-Protein Interactions

Since its invention, AFM has strongly contributed to the study of DNA-protein interactions. These interactions have a fundamental role in the regulation of several biological processes such as DNA transcription, replication, and repair. Perturbation in DNA-protein interactions could be at the origin of severe diseases, such as cancer [13]. Thus, their investigation is crucial to understand numerous cellular processes. In this sense, several methods and techniques have been developed to study such interactions. Some of them aim to identify DNA-interacting proteins (i.e., chromatin immunoprecipitation analysis-based methods [14] and electrophoretic mobility shift assays [15]), while others are used to investigate DNA-protein complex (i.e., fluorescence, electron, and atomic force microscopies).

AFM is frequently used to study DNA-protein complexes. When investigated in imaging mode, DNA and proteins need to be attached to an atomically flat surface such as mica. However, this surface gets negatively charged in aqueous medium and interrupts the binding of similarly charged molecules. Thus, the DNA attachment requires the addition of divalent cations in the imaging medium or the pretreatment of the mica surface.

These solutions necessitate finding the adequate balance of attachment forces between DNA-protein complexes and mica surface. Indeed, a strong fixation of molecules to the substrate can inhibit their function, whereas the weakness of the attachment allows it while compromising the AFM imaging.

Several studies have investigated DNA-protein complexes. Sun et al. have used the AFM to investigate the role of the single-stranded DNA-binding protein (SSB) in the interaction between the RecG DNA helicase with DNA fork [16]. According to the morphological information of the DNA-SSB-RecG complex, obtained with AFM, the authors observed that SSB enhances RecG loading efficiency onto the DNA fork. Moreover, the interaction of SSB with RecG leads to the remodeling of the latter [16].

More recently, Chen et al. have explored the interaction of the tumor suppressor protein p53 with DNA in metal ion solution using AFM [17]. The obtained results highlight the positive influence of magnesium ions on p53-DNA binding. Indeed, the authors suggest that magnesium ions significantly stimulate the binding of p53 to DNA. In addition, at high concentrations, the magnesium ions can promote p53 aggregation leading to the formation of self-assembly networks of DNA and p53 proteins [17].

Other possibility provided by the AFM technology is the measurement of the interaction forces between DNA and its interacting proteins. The experimental protocol tends to bind DNA to AFM tips via a polymer spacer, while proteins are immobilized on the surface. Multiple approach-retract cycles result in a series of force-distance measurements. Thus, unbinding events can be identified by a typical stretching profile of the polymer spacer before the point of bond rupture. Using this procedure, Bartels et al. have investigated the transcriptional regulator ExpG in the gram-negative soil bacterium *Sinorhizobium meliloti*. They explored the binding mechanism of ExpG to three promoter fragments (expA1, expG-expD1, and expE1) of the exp gene cluster. AFM force spectroscopy experiments confirmed the specific binding of ExpG to the promoter regions, with an interaction force in the range of 50 to 165 pN [18].

For more in-depth overview of the AFM achievement in molecular biology field, two detailed reviews are worthy of note [19, 20].

### 3.2. Structural and Functional Investigations of Living Cells

**Figure 2:**
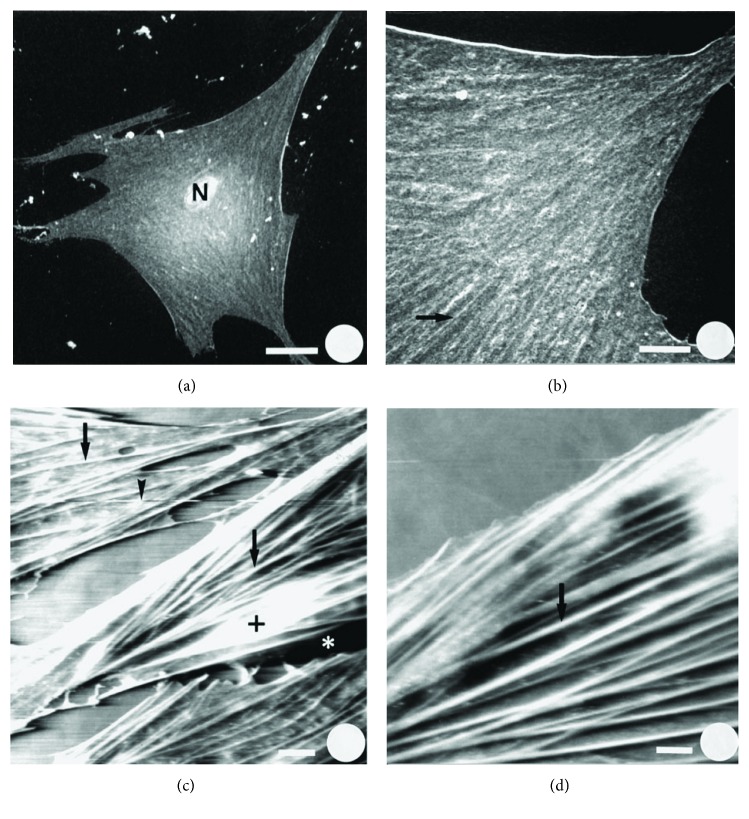
SEM and AFM images from skin fibroblasts. (a) SEM image from skin fibroblast (N: nucleus; white bar: 20 *μ*m). (b) Higher magnification of the cytoplasm (white bar: 5 *μ*m). (c) Low-magnification AFM image of fibroblast (➙: cytoskeletal fibers; ∗: AFM artifacts in the form of shadowing structures, **+**: white bumps; white bar: 10 *μ*m). (d) Higher magnification image showing parallel fibers (white bar: 5 *μ*m). Reprinted with permission from [21], copyright John Wiley and Sons.

AFM presents many advantages compared to other imaging techniques. It allows the observation of specific structures, such as the cytoskeleton (Figure 2) [21], and the study of dynamic changes in submembranous structures. Moreover, the sample preparation does not require any labeling or coating. Besides, the experiments could be performed in aqueous environment mimicking the physiological conditions [21].

Many AFM applications in mammalian cell studies have been applied in cancer cell research. Accordingly, one of these studies (Zouaoui et al. [22]) has compared the mechanical properties of highly aggressive metastatic (PC-3) and nontumoral (WPMY-1) prostate cell lines. In this sense, the viscoelasticity of single cells was measured via AFM. The results have shown a decrease in the stiffness and a less viscous behavior of the PC-3 cells, conferring to them higher deformation capacities in comparison with the WPMY-1 control cells [22].

More recently, Cascione et al. have evaluated the inhibition effects of the Rho-associated coiled-coil containing protein kinase (ROCK) on living breast cancer epithelial cells [23]. They used AFM to evaluate changes of cellular elasticity and morphometric alterations. The obtained results demonstrated that the use of ROCK inhibitor (Y-27632) increases the cell rigidity and interrupts the metastasization process via the prevention of cell migration [23].

AFM is not only used to explore tumoral cells but also to investigate numerous other cells types. For instance, Lanzicher et al. [24] have studied the biomechanical behavior of cardiac cells carrying the lamin A/C D192G mutation, which is at the origin of the development of heart failure in some DCM patients [25]. The AFM-derived mechanical properties of the cardiac cells expressing D192G mutant show an increase of cell stiffness compared to control [24]. Moreover, other studies have used AFM to understand the structural role of other cytoskeletal proteins. In this light, Tangney et al. have investigated the involvement of vinculin in the structure and function maintenance of cardiac myocytes [26]. The AFM measurements, in cardiomyocyte-specific vinculin knockout mice, have suggested that the loss of vinculin function induces a decrease in membrane cortical stiffness of cardiac myocytes [26].

Recently, Smolyakov et al. [27] have explored the biophysical properties of cardiomyocytes surface via the optimization of a novel multiparametric AFM mode. The authors have investigated the topography and mechanical properties of the cardiac lateral membrane. This novelty allows the characterization of subsarcolemmal structures, such as mitochondria and sarcomeric apparatus, identifying both Z-lines and M-bands [27].

**Figure 3:**
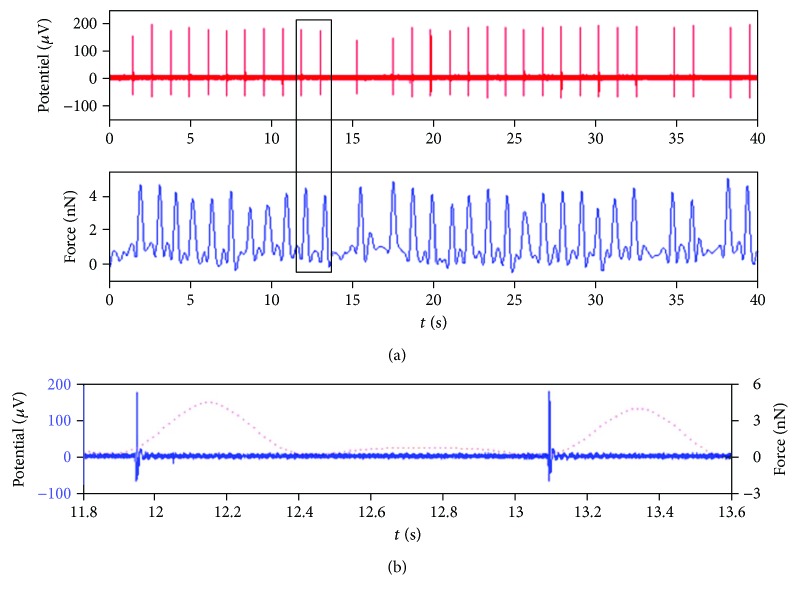
Electromechanical signals recorded from a single cardiomyocyte on the MEA chip. (a) The spike train (top) and the synchronized beating pulses (bottom). (b) Zoom on the eletromechanical signals located in the inset frame (black rectangle). Solid signals represent the recording of extracellular field potential, and the dashed ones represent the cardiomyocytes beating force. Reprinted with permission from [3], copyright 2014.

Besides, the AFM technique was combined with the microelectrode Array (MEA) to detect the electromechanical activities of cardiomyocytes as well as to characterize the effects of pharmacological drugs [3]. To do this, neonatal rat cardiomyocytes were isolated and cultured. Then, one milliliter of cell suspension was filled into a cell chamber which is an assembly of the MEA chip and glass ring fixed on its top. On day 6 of the culture, the MEA chip with cardiomyocytes was set onto the AFM. Thereafter, the cardiac cells on top of a microelectrode were positioned beneath the probe tip. When the cantilever makes contact with the cardiomyocytes and after parameter setting, the recording of extracellular field potential and the cantilever deflection detection started simultaneously (Figure 3). Of note, the cantilever deflection was converted to force data that reflects the cardiomyocyte beating force [3].

Many other achievements of AFM were realized in different fields, such as structural biology, pharmacology, and microbiology. We refer the interested readers to these valuable review articles [5, 28, 29].

## 4. The FluidFM Technology

**Figure 4:**
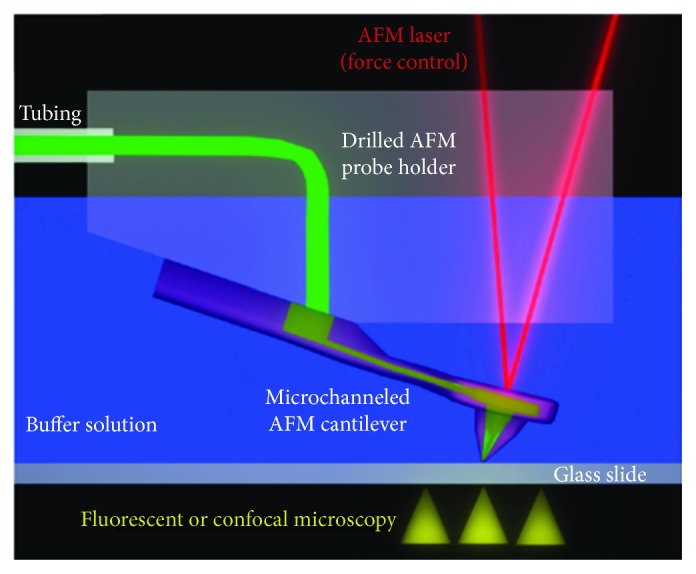
Schematic representation of FluidFM technology. This setup includes a microchanneled cantilever fixed, in a watertight way, to a drilled AFM probe-holder; overpressure can be applied in the nanofluidic channel allowing the delivery of dyes, bioactive molecules, and microorganisms through an aperture in the pyramidal AFM tip. Moreover, the solution contained into the microchannel circuit can also be used to perform electrophysiological experiments. Reprinted with permission from [6], copyright 2009 American Chemical Society.

Since its invention in the 1980s, AFM has known huge improvements, such as the development of fluidic force microscopy (FluidFM). The latter was developed by Zambelli's group (ETH Zurich, Switzerland) and consists of combining a conventional AFM with microchanneled cantilevers tightly connected to an external reservoir (Figure 4) [6]. This technology allows overcoming several limitations presented by the conventional AFM. Indeed, the application of pressure via a fluidic circuit has permitted to accomplish diverse experiments such as the quantification of cell adhesion forces, biomolecules delivery, cell injection, patch clamping, and sampling followed by analysis [8, 9, 11, 30–34].

The microfabrication of microchanneled cantilevers is based on thermal fusion of two silicon wafers to create cavities lined with silicon dioxide within the body of the silicon. Then, the cantilever microchannel enters the silicon chip and ends in an open reservoir [6]. Finally, an aperture at the apex of the cantilever tip is realized using focused ion beam milling [6]. Thus, the obtained tips confer the versatility of fluidics with the accuracy of the AFM force control. The latter allows gentle manipulation and isolation of single cells [7, 35], quantification of cell-substrate, and cell-cell interaction [8, 34], while pressure control allows quantitative manipulation of liquids and reversible immobilization of cell at the aperture edge.

### 4.1. Controlled Deposition and Injection of Biomolecules

The primary achievement brought about by FluidFM technology is the accurate delivery of bioactive substances to a single-targeted cell. In this respect, Meister et al. have performed a force-controlled delivery of fluorescent dyes into a neuroblastoma without any cell damage. The intracellular injections were performed using two different approaches. The first was the injection of 10 fL of FITC dye by a hydrostatic pressure using a very sharp probe. Following this injection, the authors observed the appearance of a fluorescence signal without significant modification in the cell volume. In addition, using the same microchanneled cantilever, three different neurons have been consecutively injected with the FITC dye, demonstrating that probes could be used for multiple manipulations without clogging [6]. The second approach was the utilization of a gentle contact procedure to stain living neuroblastoma cells. To do so, a microchannelled cantilever filled with a membrane-permeant dye was placed on cells and left in gentle contact until the dye diffused into the cytoplasm.

In the same study, Meister et al. have shown the ability of the FluidFM system to accurately deliver molecules into the selected subcellular structure. For this purpose, the region of interest was first scanned. Then, based on the obtained topographical information, the authors were able to characterize structures that are hardly accessible to the optical microinjection systems. In this study, a varicosity-like structure, localized in the middle of two connected neuroblastomas, was successfully injected with a membrane-permeant dye called acridine orange [6].

Later, Guillaume-Gentil et al. have demonstrated the versatility of the FluidFM-based injection system by simultaneously filling the cantilever microchannel with dyes or bioactive molecules [7, 33]. First, the authors have injected into HeLa cell nuclei a nonpermeable dye, the dextran-tetramethylrhodamine. The acquired fluorescence images show that the injected dye remained retained into the nucleus [33]. Moreover, using the same approach, a plasmid DNA encoding the green fluorescent protein (GFP) was injected into the cell nuclei. Two hours later, a transient expression of GFP was detected [33]. Second, the same team focused on the optimization of a force-controlled protocol aiming to selectively isolate single cells from confluent layers [7]. The fluidic force microscopy was used to isolate the selected cells via a localized trypsinization. The cell was then caught through gentle aspiration allowing its transfer on the desired emplacement [7].

Furthermore, FluidFM was also used to deliver microorganisms. In this sense, Stiefel et al. have studied the infection mode of VACV virion by loading its fluorescent form into a microchanneled cantilever [36]. Thus, a small number of these virions (from 1 to 12) were deposited one-by-one in a controlled manner onto single HeLa cells [36].

### 4.2. Spatial Manipulation of Living Cells

As stated before, thanks to the fluidic circuit integrated in the FluidFM system, the manipulator can accurately manipulate microobjects. These objects could be reversibly immobilized at the cantilever aperture by application of negative pressure. Then, the trapped object can be repositioned on the desired substrate and released by application of positive pressure. By this principle, Dörig et al. have displaced mammalian cells (neurons), yeasts (*Saccharomyces cerevisiae*), and bacteria (*Escherichia coli*). For instance, *Saccharomyces cerevisiae* was manipulated by moving the cantilever in contact mode under AFM force feedback; afterward, a negative pressure was applied in the fluidic circuit. Consequently, the yeasts were immobilized on the microchannel aperture. Finally, the cells were displaced and released to the desired new position by a short positive pressure. As was described for the injection experiments, serial manipulations were possible using the same cantilever [32].

Later, based on their IR specific fluorescence, single bacteriochlorophyll-expressing bacteria were successfully isolated using the FluidFM technology [35]. More recently, Guillaume-Gentil et al. have extended the pick-and-place technique to mammalian cells [7].

### 4.3. Adhesion Force Quantification

Cell adhesion is the physiological process involving highly regulated interactions, where the cells interact with each other or their substrate. In mammalian cells, adhesion is implicated in numerous cellular functions such as differentiation, tissue development, and inflammation.

FluidFM offers several possibilities to study adhesion at the single-molecular level. Potthoff et al. have developed FluidFM-based single-cell force spectroscopy (SCFS) by substituting the conventional cell trapping cantilever chemistry by underpressure immobilization [34]. Using this system, the authors have studied the adhesion of yeasts and mammalian cells by performing serial and dynamic long-term adhesion measurements [34]. *C. albicans* is the first experimental model used in this study. Based on the force-distance curve, obtained during the detachment of *C. albicans* from a hydrophobic dodecyl phosphate surface, the maximal adhesion force and adhesion work have reached  43 nN and  8.10^−15^J, respectively. The FluidFM-based SCFS allows the serial measurement of around 200 yeast cells with the same probe [34]. Moreover, the authors have extended the applicability of this system to HeLa cells, which have an adhesion force to uncoated glass substrate of about 470 ± 130 nN at 37°C [34]. The same team has thereafter investigated the adhesion force of *Escherichia coli* and *Streptococcus pyogenes* using FluidFM. The adhesion force of *Escherichia coli* from polydopamine is nearly 4–8 nN [8].

Recently, Cohen et al. have used FluidFM-based SCFS to compare both homotypic (between MCF7 breast cancer cell line) and heterotypic adhesion forces (between MCF10A breast cancer cells and nontumorigenic HS5 cells). Cell adhesion forces were measured using short (contact duration: 1–50 s) and long-term (contact duration: 30–60 min) adhesion protocols. At short contact duration, the results reported similar adhesion forces in homotypic and heterotypic conditions, while they differ at longer contact period [37].

Overall, all the cited studies have demonstrated the added values of FluidFM in the investigation of microbial adhesion, cell-substrate, and cell-cell interactions. Indeed, in comparison with the conventional AFM, which requires the utilization of chemical treatments to immobilize the cells on the cantilever, FluidFM uses a physical process based on underpressure immobilization of the cells. Thus, we prevent the introduction of biases on the adhesion force measurements due to the potential modification of the cell physiology related to the chemical treatments. Moreover, in contrast to the irreversible chemical fixation of the cells, the underpressure immobilization is a reversible process that allows a multiple manipulation of the same cell.

### 4.4. Cellular Electrophysiology

Ion channels are membrane transporters responsible for the passage of ions through cell membranes according to the direction of their electrochemical gradients. Dysfunction of these proteins, also called channelopathies, was associated with the occurrence of several human disorders such as cardiac arrhythmias and neurodegenerative diseases [38–40]. Hence, ion channels become one of the most important molecular targets for several classes of drugs.

In this context, numerous electrophysiology techniques were developed to study the biophysical and pharmacological properties of ion channels. The patch clamp technology is considered as the gold standard technique in this field. It relies on clamping the voltage across a portion of the cell membrane by placing a glass micropipette on the cell. A small negative pressure is applied to obtain a high electric resistance known as gigaseal. Then, additional suction will rupture the membrane in the pipet providing electrical access to the cytoplasm, this configuration is called whole-cell configuration.

**Figure 5:**
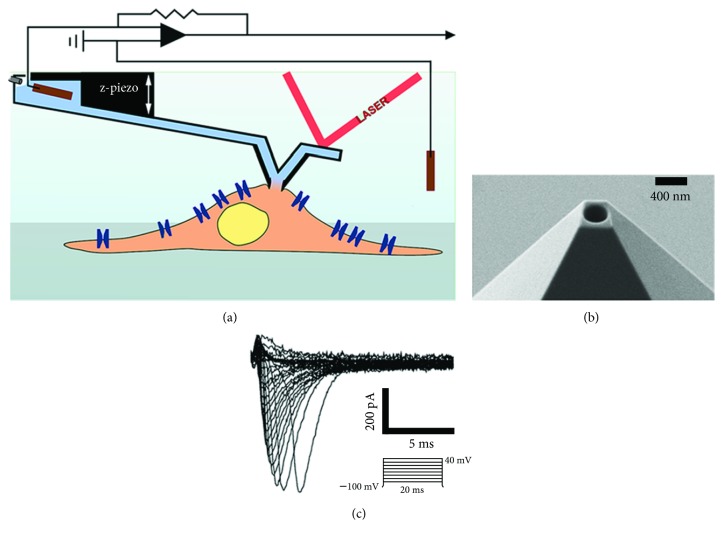
pc-FluidFM technology. (a) Schematic representation of the pc-FluidFM. (b) An example of pyramidal tip used for the patch clamp experiments. (c) Representative traces of the recorded I_Na_ current from HEK 293 cells. Adapted with permission from [9], copyright 2015 American Chemical Society.

Recently, Ossola et al. have combined the patch clamp technique to AFM via the FluidFM technology (pc-FluidFM) (Figure 5) [9]. This combination allows the study of ion channels function, while simultaneously controlling the applied force on the investigated cell. Indeed, using two different cell models (HEK293, and adult mice cardiomyocytes), the authors have recorded the fast I_Na_ current mediated via the cardiac sodium channel Na_v1.5_ (Figure 5). pc-FluidFM takes advantage of the AFM force control to obtain gigaseals and to record ionic currents even from contracting cardiac cells. Moreover, in contrast to conventional patch clamp, the authors have demonstrated the ability of pc-FluidFM to perform serial patch clamping experiments using the same probe.

On the other hand, these authors have used pc-FluidFM as an injection tool. Indeed, after obtaining the whole-cell configuration, the solution contained in the microfluidic circuit can diffuse into the cytoplasm of the studied cell. Using this principle, the authors have carried out serial injections of lucifer yellow dye in freshly isolated cardiomyocytes without any morphological changes at the end of the injection experiment [9].

One of the major limitations of the pc-FluidFM technology is the low electric resistance obtained during the seal formation (usually under 100 MΩ versus 1G Ω, or more, for the optimal gigaseal configuration). As a possible explanation for the low-resistance seals, the authors pointed the square section and small height depth of the FluidFM tips which impedes the building of gigaohmic resistances. Therefore, an increased noise level and current leakage are observed. The latest combined with the high resistance and capacitance of the FluidFM probe leads to a partial clamp of the transmembrane voltage which strongly affects the measured biophysical parameters of ion channels.

### 4.5. Single-Cell Extraction and Analysis

Single-cell variability is a basic characteristic of multicellular organisms. Among any specific tissue, we can find a heterogeneous population of cells characterized by different gene expression profiles. The development of single-cell analysis methods, such as flow cytometry, mass spectrometry-based approaches, and single-cell RNA sequencing, has revealed that cell heterogeneity is involved in several physiological processes [41].

For single-cell metabolic studies, several MS-based methods have been developed. These methods are based on the ionization of single cells, followed by the extraction of their intracellular contents. For example, Jansson et al. have characterized the single-cell heterogeneity of Langerhans islets using microscopy-guided single-cell matrix-assisted laser desoption/ionizaton time-of-flight mass spectrometry (MALDI) [42]. The authors have analyzed more than 3000 cells, demonstrating the ability of this system to investigate metabolic heterogeneity at the single-cell level. However, such methods usually require the extraction of the analyzed cells from their physiological environment. In consequence, the isolation of the cells from their neighborhood may influence their physiological function [43, 44].To overcome this limitation, the FluidFM technology offers new possibilities in the single-cell analysis field. In this sense, Guillaume-Gentil et al. have used this technology to analyze single molecules extracted from different cell compartments [11]. In this aim, FluidFM probes are optically placed on the target cell. Then, force spectroscopy is initiated to access into the desired cell compartment. Once the probe is inserted, a negative pressure is applied through the cantilever microchannel to extract the cellular content. This procedure was realized on cultured HeLa cells expressing GFP and resulted a significant decrease in GFP intensity in nucleus and cytoplasm. Moreover, to further demonstrate the selective extraction of cell compartment, cell nuclei were labeled with two different fluorescent markers, mRuby-NLS and FITC-dextran. For both markers, the extraction procedure induced a decrease of nuclear fluorescence compared to cytoplasm [11]. These results demonstrated the ability of FluidFM to selectively extract cell compartment contents without any cell damage. Indeed, after extraction of volumes up to 4.0 pL from the cytoplasm and 0.6 pL from nucleus, the majority of cells remained viable. The extraction of larger volumes induced cell death [11].

After the extraction procedure, the extracts were analyzed using three different approaches: electron microscopy and biochemical and transcriptional analyses. As an example, we describe here the results of the study of cytoplasmic extracts. The authors have analyzed between 0.6 and 0.7 pL of these extracts for the GFP, beta-actin, and beta-2-microglobulin transcripts. Ninety percent of the analyzed samples showed the expression of at least one of these genes.

On the other hand, the same analysis procedure was applied to the nucleus content. This compartment was more challenging compared with the cytoplasm. Indeed, gene transcripts were not detected in the extracted volumes (between 0.2 to 0.5 pL), while their detection was possible with a volume of 0.7 pL. Furthermore, the comparison of nuclear and cytoplasmic transcripts revealed a higher detection in the cytoplasm.

**Figure 6:**
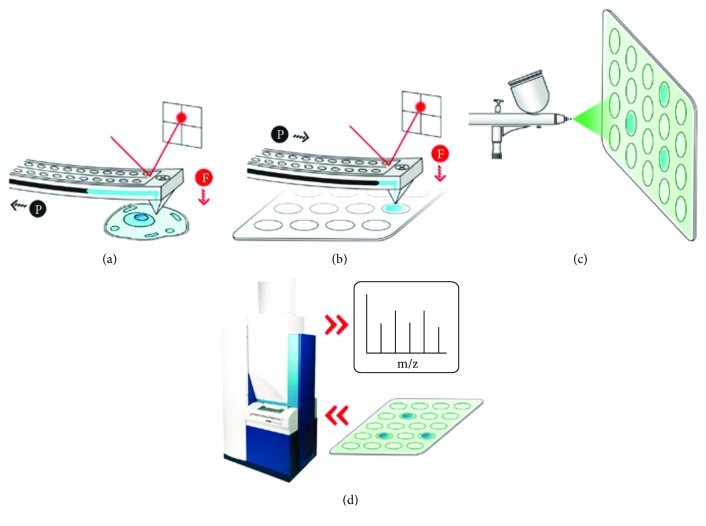
Schematic representation of the experimental procedure of the single-cell metabolic analysis using FluidFM and MALDI-TOF mass spectrometry. (a) Metabolite sampling using FluidFM. (b) Distribution of the cytoplasmic extract onto a selected MAMS spot. (c) Spraying of the 9AA matrix. (d) Acquisition of MS spectra. Adapted with permission from [31], copyright 2017 American Chemical Society.

In a more recent study, Guillaume-Gentil et al. have analyzed metabolite extracts from single live cells via the FluidFM technology coupled to a chip-based mass spectrometry [31]. First, the authors have extracted few picoliter (0.8 to 2.7 pL) of the cytoplasm content from HeLa cells. Once the desired amount of extracts was collected, the cantilever probe was placed onto the selected spot of microarrays for mass spectrometry with force-control (Figure 6). Following the release of these extracts, a MALDI-TOF analysis was realized. This analysis revealed the presence of 20 different metabolites including ribonucleotides, activated sugars, amino acids, and glutathione. As a conclusion to this work, this study has demonstrated the ability of this method to analyze cytoplasmic metabolites from single cells under their physiological environment [31].

## 5. Conclusion

Since their invention, AFM and FluidFM have responded to many biological issues in several research areas (i.e., structural biology, microbiology, molecular biology, and biophysics). Indeed, FluidFM is currently used to characterize cells structure, manipulate and inject single cells with dyes and bioactive molecules, extract and analyze metabolites from specific cell compartments, and simultaneously record electrical and contractile cells activities. Henceforth, if the future development of FluidFM is used as a combination of different operation modes, it might help to simultaneously investigate the structural and molecular as well as the functional effects of bioactive molecules before and after their delivery, especially at the multicellular scale.

## References

[1] Binnig G., Rohrer H., Gerber C., Weibel E. (1982). Surface studies by scanning tunneling microscopy. *Physical Review Letters*.

[2] Binnig G., Quate C. F., Gerber C. (1986). Atomic force microscope. *Physical Review Letters*.

[3] Tian J., Tu C., Huang B., Liang Y., Zhou J., Ye X. (2018). Study of the union method of microelectrode array and AFM for the recording of electromechanical activities in living cardiomyocytes. *European Biophysics Journal*.

[4] Blaue C., Kashef J., Franz C. M. (2017). Cadherin-11 promotes neural crest cell spreading by reducing intracellular tension–mapping adhesion and mechanics in neural crest explants by atomic force microscopy. *Seminars in Cell & Developmental Biology*.

[5] Formosa-Dague C., Duval R. E., Dague E. (2018). Cell biology of microbes and pharmacology of antimicrobial drugs explored by atomic force microscopy. *Seminars in Cell & Developmental Biology*.

[6] Meister A., Gabi M., Behr P. (2009). FluidFM: combining atomic force microscopy and nanofluidics in a universal liquid delivery system for single cell applications and beyond. *Nano Letters*.

[7] Guillaume-Gentil O., Zambelli T., Vorholt J. A. (2014). Isolation of single mammalian cells from adherent cultures by fluidic force microscopy. *Lab Chip*.

[8] Potthoff E., Ossola D., Zambelli T., Vorholt J. A. (2015). Bacterial adhesion force quantification by fluidic force microscopy. *Nanoscale*.

[9] Ossola D., Amarouch M. Y., Behr P., Voros J., Abriel H., Zambelli T. (2015). Force-controlled patch clamp of beating cardiac cells. *Nano Letters*.

[10] Ossola D., Dorig P., Voros J., Zambelli T., Vassalli M. (2016). Serial weighting of micro-objects with resonant microchanneled cantilevers. *Nanotechnology*.

[11] Guillaume-Gentil O., Grindberg R. V., Kooger R. (2016). Tunable single-cell extraction for molecular analyses. *Cell*.

[12] Meyer G., Amer N. M. (1988). Novel optical approach to atomic force microscopy. *Applied Physics Letters*.

[13] Engin H. B., Kreisberg J. F., Carter H. (2016). Structure-based analysis reveals cancer missense mutations target protein interaction interfaces. *PLoS One*.

[14] Orlando V. (2000). Mapping chromosomal proteins in vivo by formaldehyde-crosslinked-chromatin immunoprecipitation. *Trends in Biochemical Sciences*.

[15] Hellman L. M., Fried M. G. (2007). Electrophoretic mobility shift assay (EMSA) for detecting protein-nucleic acid interactions. *Nature Protocols*.

[16] Sun Z., Tan H. Y., Bianco P. R., Lyubchenko Y. L. (2015). Remodeling of RecG helicase at the DNA replication fork by SSB protein. *Scientific Reports*.

[17] Chen Y., Gao T., Wang Y., Yang G. (2017). Investigating the influence of magnesium ions on p53–DNA binding using atomic force microscopy. *International Journal of Molecular Sciences*.

[18] Bartels F. W., Baumgarth B., Anselmetti D., Ros R., Becker A. (2003). Specific binding of the regulatory protein ExpG to promoter regions of the galactoglucan biosynthesis gene cluster of Sinorhizobium meliloti–a combined molecular biology and force spectroscopy investigation. *Journal of Structural Biology*.

[19] Kasas S., Dietler G. (2018). DNA-protein interactions explored by atomic force microscopy. *Seminars in Cell & Developmental Biology*.

[20] Beckwitt E. C., Kong M., Van Houten B. (2018). Studying protein-DNA interactions using atomic force microscopy. *Seminars in Cell & Developmental Biology*.

[21] Braet F., Seynaeve C., De Zanger R., Wisse E. (1998). Imaging surface and submembranous structures with the atomic force microscope: a study on living cancer cells, fibroblasts and macrophages. *Journal of Microscopy*.

[22] Zouaoui J., Trunfio-Sfarghiu A. M., Brizuela L. (2017). Multi-scale mechanical characterization of prostate cancer cell lines: relevant biological markers to evaluate the cell metastatic potential. *Biochimica et Biophysica Acta (BBA) – General Subjects*.

[23] Cascione M., De Matteis V., Toma C. C., Pellegrino P., Leporatti S., Rinaldi R. (2017). Morphomechanical and structural changes induced by ROCK inhibitor in breast cancer cells. *Experimental Cell Research*.

[24] Lanzicher T., Martinelli V., Puzzi L. (2015). The cardiomyopathy lamin A/C D192G mutation disrupts whole-cell biomechanics in cardiomyocytes as measured by atomic force microscopy loading-unloading curve analysis. *Scientific Reports*.

[25] Sylvius N., Bilinska Z. T., Veinot J. P. (2005). In vivo and in vitro examination of the functional significances of novel lamin gene mutations in heart failure patients. *Journal of Medical Genetics*.

[26] Tangney J. R., Chuang J. S., Janssen M. S. (2013). Novel role for vinculin in ventricular myocyte mechanics and dysfunction. *Biophysical Journal*.

[27] Smolyakov G., Cauquil M., Severac C. (2017). Biophysical properties of cardiomyocyte surface explored by multiparametric AFM. *Journal of Structural Biology*.

[28] Morton K. C., Baker L. A. (2014). Atomic force microscopy-based bioanalysis for the study of disease. *Analytical Methods*.

[29] Shi Y., Cai M., Zhou L., Wang H. (2018). The structure and function of cell membranes studied by atomic force microscopy. *Seminars in Cell & Developmental Biology*.

[30] Guillaume-Gentil O., Potthoff E., Ossola D., Franz C. M., Zambelli T., Vorholt J. A. (2014). Force-controlled manipulation of single cells: from AFM to FluidFM. *Trends in Biotechnology*.

[31] Guillaume-Gentil O., Rey T., Kiefer P. (2017). Single-cell mass spectrometry of metabolites extracted from live cells by fluidic force microscopy. *Analytical Chemistry*.

[32] Dörig P., Stiefel P., Behr P. (2010). Force-controlled spatial manipulation of viable mammalian cells and micro-organisms by means of FluidFM technology. *Applied Physics Letters*.

[33] Guillaume-Gentil O., Potthoff E., Ossola D., Dorig P., Zambelli T., Vorholt J. A. (2013). Force-controlled fluidic injection into single cell nuclei. *Small*.

[34] Potthoff E., Guillaume-Gentil O., Ossola D. (2012). Rapid and serial quantification of adhesion forces of yeast and mammalian cells. *PLoS One*.

[35] Stiefel P., Zambelli T., Vorholt J. A. (2013). Isolation of optically targeted single bacteria by application of fluidic force microscopy to aerobic anoxygenic phototrophs from the phyllosphere. *Applied and Environmental Microbiology*.

[36] Stiefel P., Schmidt F. I., Dörig P. (2012). Cooperative vaccinia infection demonstrated at the single-cell level using FluidFM. *Nano Letters*.

[37] Cohen N., Sarkar S., Hondroulis E., Sabhachandani P., Konry T. (2017). Quantification of intercellular adhesion forces measured by fluid force microscopy. *Talanta*.

[38] Abriel H., Syam N., Sottas V., Amarouch M. Y., Rougier J. S. (2012). TRPM4 channels in the cardiovascular system: physiology, pathophysiology, and pharmacology. *Biochemical Pharmacology*.

[39] Abriel H., Zaklyazminskaya E. V. (2013). Cardiac channelopathies: genetic and molecular mechanisms. *Gene*.

[40] Amarouch M. Y., Abriel H. (2015). Cellular hyper-excitability caused by mutations that alter the activation process of voltage-gated sodium channels. *Frontiers in Physiology*.

[41] Lidstrom M. E., Konopka M. C. (2010). The role of physiological heterogeneity in microbial population behavior. *Nature Chemical Biology*.

[42] Jansson E. T., Comi T. J., Rubakhin S. S., Sweedler J. V. (2016). Single cell peptide heterogeneity of rat islets of Langerhans. *ACS Chemical Biology*.

[43] Wojtusciszyn A., Armanet M., Morel P., Berney T., Bosco D. (2008). Insulin secretion from human beta cells is heterogeneous and dependent on cell-to-cell contacts. *Diabetologia*.

[44] Benninger R. K. P., Hodson D. J. (2018). New understanding of *β*-cell heterogeneity and in situ islet function. *Diabetes*.

